# Whistle repertoire and structure reflect ecotype distinction of pantropical spotted dolphins in the Eastern Tropical Pacific

**DOI:** 10.1038/s41598-023-40691-8

**Published:** 2023-08-18

**Authors:** Manali Rege-Colt, Julie N. Oswald, Joelle De Weerdt, Jose David Palacios-Alfaro, Maia Austin, Emma Gagne, Jacqueline Maythé Morán Villatoro, Catherine Teresa Sahley, Gilma Alvarado-Guerra, Laura J. May-Collado

**Affiliations:** 1 Biology Department, University of Vermont, Burlington, VT USA; 2 Scottish Oceans Institute, Sea Mammal Research Unit, University of St. Andrews, St. Andrews, KY168LB UK; 3 Association ELI-S, Education, Liberté, Indépendance - Scientifique, Allée de Verdalle 39, 33470 Gujan-Mestras, France; 4 Vrije Universiteit Brussel (VUB), Pleinlaan 2, 1050 Brussels, Belgium; 5 Panacetacea.Org, Saint Paul, MN USA; 6 Energía del Pacífico, Ltda. de C.V. /Invenergy LLC, Municipality of Acajutla, Sonsonate, El Salvador; 7 Environmental Resources Management, Inc., Breckville, OH USA; 8 Instituto para el Crecimiento Sostenible de la Empresa (ICSEM), C/Hogar Padre Vito Guarato, B1, San Salvador, El Salvador; 9 Smithsonian Tropical Research Institute, Panama, Panama

**Keywords:** Animal behaviour, Behavioural ecology, Marine mammals

## Abstract

The pantropical spotted dolphin in the Eastern Tropical Pacific (ETP) is found in two genetically and phenotypically diverged ecotypes, coastal and offshore. These habitats have distinct acoustic characteristics, which can lead to the evolution of distinct acoustic communication. Whistles are sounds widely used by dolphins to mediate species and individual recognition and social interactions. Here, we study the whistle acoustic structure and repertoire diversity of offshore and coastal pantropical spotted dolphins. Our results show that there is significantly more within- and across-group variation in whistle fundamental frequency between ecotypes than between offshore groups and between coastal groups. A Random Forest classification analysis performed with an accuracy of 83.99% and identified duration, peak and minimum frequency as the most informative variables for distinguishing between ecotypes. Overall, coastal spotted dolphins produced significantly shorter whistles that were significantly lower in frequency (peak, minimum and maximum, and start and end) than offshore dolphins. Ecotypes produced whistle repertoires that were similar in diversity, but different in contour composition, with the coastal ecotype producing more upsweep whistles than offshore dolphins. The results of this study suggest that acoustic adaptations to coastal and offshore environments could be important contributors to intraspecific variation of dolphin whistle repertoires.

## Introduction

Dolphins produce narrowband and frequency modulated sounds called whistles. These sounds vary in duration and have fundamental frequencies ranging between 1 and 75 kHz^1–3^. Whistles are important in dolphin communication, as they convey information about individual identity, behavioral state, and the environment^4,5^. They are also used for group cohesion, coordination of activities, and maintaining communication when separated^6,7^. In Indo-Pacific bottlenose dolphins (*Tursiops aduncus*)^5,8–11^, striped dolphins (*Stenella coeruleoalba*)^12^, Guiana dolphins (*Sotalia guianensis*)^13^, bottlenose dolphins (*Tursiops truncatus*)^2^, and short beaked common dolphins (*Delphinus delphis*)^14^), within species variation in whistle frequency and duration has been explained in the context of geographical and behavioral constraints, and ecological adaptations. In contrast, factors contributing to variations in dolphin whistle repertoires (all the different whistle contours an individual, group, population or species makes), and the diversity and complexity of these repertoires are less understood but may be dependent on group size and strength of conspecific associations^15^, and culture^16^.

The pantropical spotted dolphin (*Stenella attenuata*) occurs in tropical and subtropical regions between 30–40 degrees north and 20–40 degrees south^17,18^. Despite the wide distribution of this species, little information exists regarding their whistle repertoire or structure. Studies in Brazil and the Eastern Tropical Pacific (ETP) have described spotted dolphin whistles as consisting primarily of convex contours with fundamental frequencies ranging from 8.2 to 31.1 kHz^19–25^. In the ETP, pantropical spotted dolphins are classified into coastal (*S. attenuata graffmani*) and offshore (*S. attenuata attenuata*) ecotypes, with the latter divided into northeastern and western-southern stocks^26,27^. This classification is supported by phenotypic differences in skull morphology, body size, and spotting patterns^28^, genomic data^27,29,30^ and behavioral data (i.e., group size)^26^. The larger coastal ecotype is heavily spotted^31^, and lives within 200 km of the coast of Central America in groups of up to 50 individuals^26,32^. In contrast, the offshore ecotype is lightly spotted and lives in pelagic habitats in groups of hundreds of individuals^31^. Molecular evidence suggests that the coastal and offshore ecotypes are genetically distinct^29^ and that coastal populations throughout Central America are genetically structured^27^, supporting recognition of ecotypes as separate conservation and management units.

The ecotype distinction of pantropical spotted dolphins warrants investigation into whether differences in habitat translate into their acoustic repertoire. Coastal and offshore dolphins experience different acoustic environments^33^. The physical characteristics of these acoustic environments can directly affect propagation of acoustic signals and drive changes in signal structure to overcome such constraints^33–35^, as predicted by the “Acoustic Adaptation Hypothesis” (AAH)^36^. The AAH states that in response to environmental constraints, animals adjust their signals to maximize signal propagation and experience less attenuation and degradation. Genetic differentiation between spotted dolphin ecotypes and subsequent habitat specialization can be further reinforced by this acoustic repertoire differentiation^37–39^.

In this study we compare the whistle acoustic structure and repertoire diversity of offshore and coastal pantropical spotted dolphins. Our objectives are three-fold. This study (1) assesses the ability to differentiate between coastal and offshore dolphin whistles based on fundamental frequency contours, (2) explores how ecotype whistles are distinct in their acoustic structure using standard parameter measurements, and (3) compares the composition and diversity of whistle repertoires between ecotypes. We hypothesize that given ecotype distinction, overall repertoire diversity will differ, and that contour composition and the temporal and frequency characteristics of whistles should reflect acoustic adaptations to their distinct soundscapes^36^. This study provides insights on the potential role of habitat specialization on dolphin whistle repertoire, with applications to identification of spotted dolphin ecotypes in passive acoustic monitoring efforts in the ETP.

## Results

**Figure 1:**
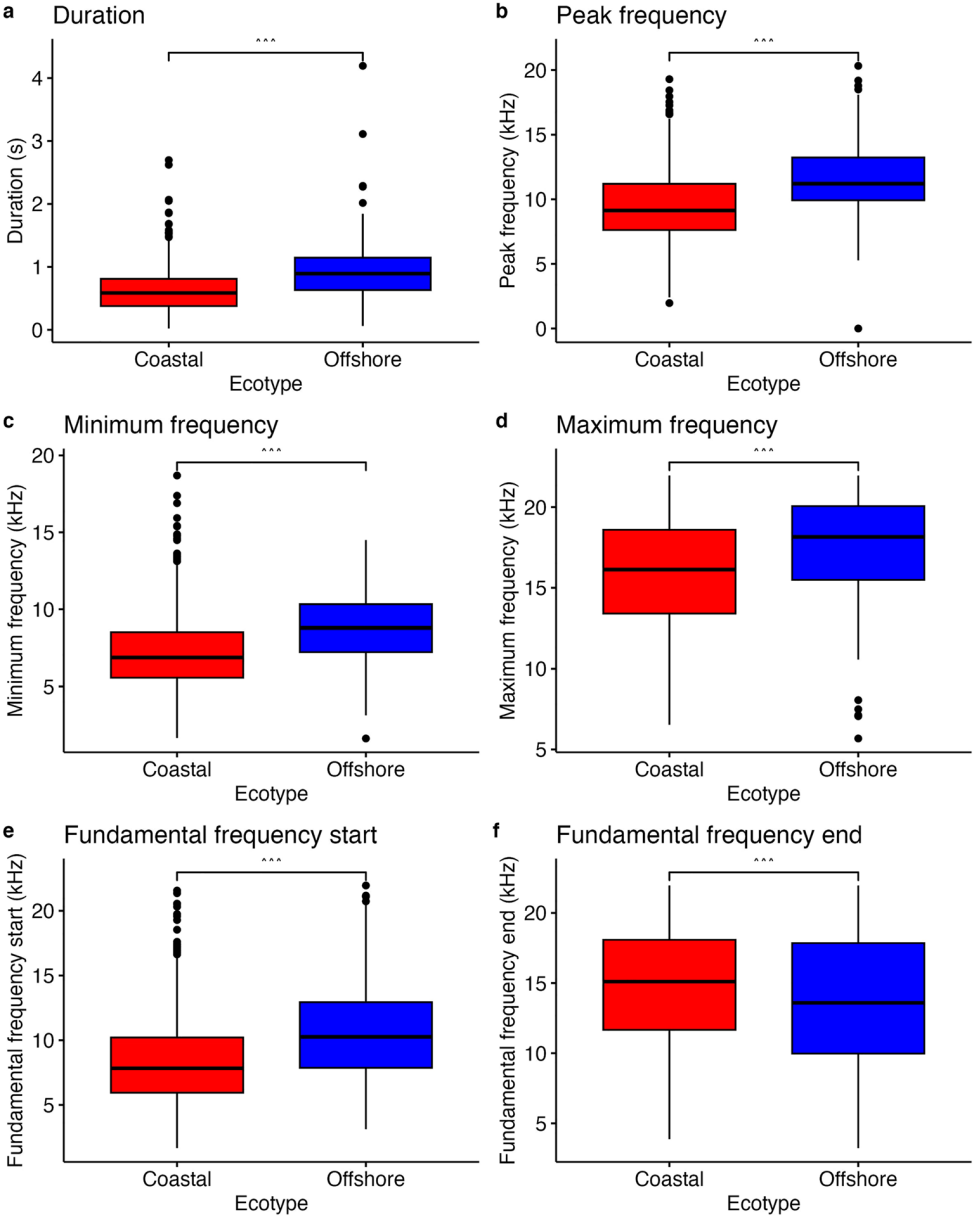
Pantropical spotted dolphin whistle variation in frequency and duration between offshore and coastal ecotypes. Differences between ecotypes are statistically significant based on a Mann–Whitney U test (α = 0.05) with every parameter difference p-value < 0.00001. Each boxplot shows the first and third quartiles, medians, and standard error ((**a**) Duration, (**b**) Peak frequency, (**c**) Minimum frequency, (**d**) Maximum frequency, (**e**) Fundamental frequency start, (**f**) Fundamental frequency end).

A total of 1312 whistles (coastal = 657, offshore = 655) were extracted from 11.2 h of total recording effort (Supp. Table S1) and imported into Luscinia. Due to differences in recording sampling rate, whistles were subsampled to include only whistles with fundamental frequencies entirely below 22.05 kHz, resulting in a subsample of 958 whistles (coastal = 492, offshore = 466). Descriptive statistics of whistle acoustic structure by ecotype are shown in Supplementary Table S2. We found significant differences between ecotypes in all acoustic variables except delta frequency (duration: W = 84,036, p < 0.00001; peak frequency: W = 84,028, p < 0.00001; minimum frequency: W = 88,602, p < 0.00001; maximum frequency: W = 111,328, p < 0.00001; fundamental frequency start: W = 98,189, p < 0.00001; fundamental frequency end: W = 175,591, p = 0.00004; delta frequency: W = 153,594, p = 0.928). Overall, offshore pantropical spotted dolphins produced whistles that were longer and higher in most frequency variables than those produced by coastal pantropical spotted dolphins (Fig. 1).

### Ecotype classification

**Figure 2:**
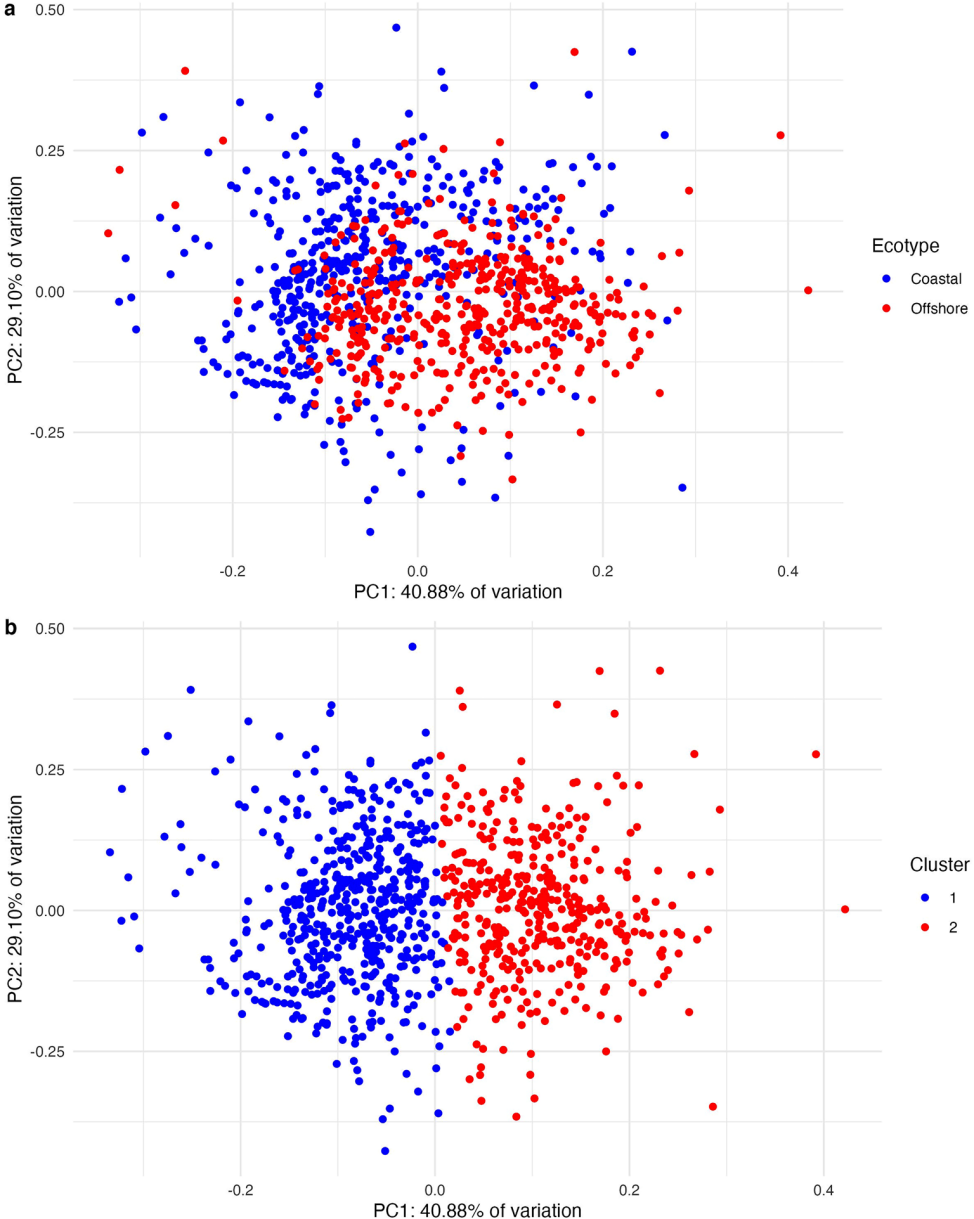
Non-metric multidimensional scaling of pantropical spotted coastal and offshore dolphin whistles (**a**) based on a dissimilarity matrix in which the relative distance between points is representative of their (dis)similarity and a (**b**) K-medoids cluster analysis in which the combined dataset of coastal and offshore whistles was blindly clustered into k = 2 groups based on their dissimilarity.

The Non-Metric Multidimensional Scaling (NMMDS) analysis shows that the repertoires of the two ecotypes overlap based on contour fundamental frequency but have potential for ecotype classification (Fig. 2a). The k-medoids cluster analysis classified whistle contours by ecotype with a 70.06% accuracy (Table 1). A total of 69.91% of the variation between ecotypes was explained by two dimensions (Fig. 2b).

**Table 1:** Results of pantropical spotted dolphin whistle classification by ecotype of the confusion matrix from k-medoids clustering analysis (accuracy of 70.06%) and confusion matrix of random forest model performance on test data (accuracy of 83.99%).

Prediction	Reference	
	Coastal	Offshore
K-medoids clustering analysis		
Coastal	374	167
Offshore	118	293
Random forest model		
Coastal	131	27
Offshore	22	126

The Multivariate Response Permutation Procedure (MRPP) shows that there are significant differences in whistle fundamental frequency among offshore (empirical δ = 0.235, p = 0.0001) and coastal (empirical δ = 0.245, p = 0.0001) groups, and between ecotypes (empirical δ = 0.244, p = 0.0001) (Supp. Table S3). However, the effect size between ecotypes is greater (a = 0.0493) than the effect size between offshore (a = 0.0383) and coastal (a = 0.00901) groups. Furthermore, the Multivariate Dispersion analyses, indicates that within-group variability of each offshore group was not significantly different (p = 0.836, F = 0.176) and similar results were found for the coastal groups (p = 0.464, F = 0.530). In contrast, within-group variability between ecotypes was significantly different (p = 0.0046, F = 8.346) (Supp. Table S3).

The random forest (RF) model classified whistle contours by ecotype with 83.99% accuracy (95% CI from 79.39–87.91%) (Table 1). After a 67–33% split of the data, 626 whistles (313 whistles per ecotype) were included in the training set and 306 whistles (153 whistles per ecotype) created the test set. The final RF model considered two random predictor variables (mtry = 2) at each node split out of the seven possible predictors and grew 300 trees. The model had an out of bag (OOB) error rate of 16.61% and a kappa statistic of 0.68. Using the kappa statistic scale as per Landis and Koch^40^, the model is in “substantial” agreement with the true population.

Mean Decrease in Accuracy (MDA) and Mean Decrease in Gini (MDG) values showed that duration, peak frequency (PF), and minimum frequency (MF), in this order were the predictor variables that generated the most accurate classification of whistles by ecotype (Table 2). The MDA and MDG results mean that the permutation of the previously listed parameters in the model resulted in the highest decrease in accuracy and node homogeneity. Partial Dependence Plots (PDP) provided insight into how the RF classified whistles using the top three most important predictors (Supp. Fig. S3). The duration PDP shows that whistles with a duration of at most ~ 0.6 s have a maximum likelihood of being accurately classified as the coastal ecotype, while whistles with a duration of at least 1 s are likely to be classified as the offshore ecotype. Meanwhile, the peak frequency PDP shows that whistles with peak frequency values less than or equal to 10 kHz are most likely to be classified as the coastal ecotype, while offshore whistles can be classified as having peak frequency greater than or equal to 10 kHz. The minimum frequency PDP shows that whistles with minimum frequency less than or equal to 6 kHz are most likely to be classified as the coastal ecotype while offshore whistles are accurately classified with minimum frequency above 10 kHz. These results are in accordance with the non-parametric analysis of each of these whistle variables described above.

**Table 2:** Mean decrease in accuracy (MDA) and mean decrease in Gini (MDG) of the predictor variables from the RF model in which duration, peak frequency and minimum frequency hold the most importance when classifying pantropical spotted dolphin whistles by ecotype.

Predictor variables	Mean decrease accuracy	Mean decrease Gini
Duration (s)	45.32	70.53
Peak frequency (kHz)	36.66	57.09
Minimum frequency (kHz)	31.84	47.13
Delta frequency (kHz)	24.25	31.48
Maximum frequency (kHz)	22.94	32.28
Fundamental frequency end (kHz)	22.64	35.43
Fundamental frequency start (kHz)	20.08	38.55

### Repertoire analysis

**Figure 3:**
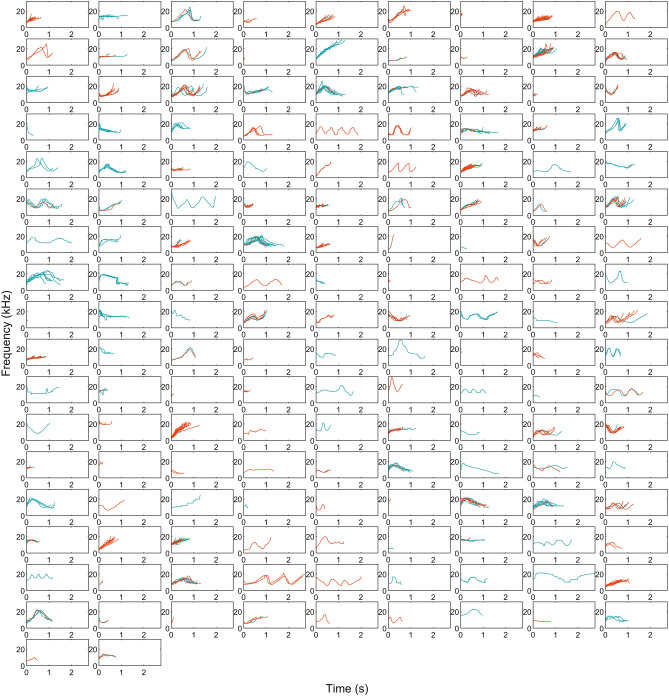
The ARTwarp categorization of the combined offshore and coastal ecotype whistles (n = 916) using 96% vigilance. The categorization resulted in 223 whistle categories. Each window is a neuron that represents the spectrogram of a category that the neural network has created with time on the x-axis (0–5 s) and frequency on the y-axis (0–20 kHz). Within each neuron are the contours that were categorized as belonging to that neuron. Coastal ecotype whistles contours are shown in red and offshore ecotype whistle contours are shown in blue.

**Figure 4:**
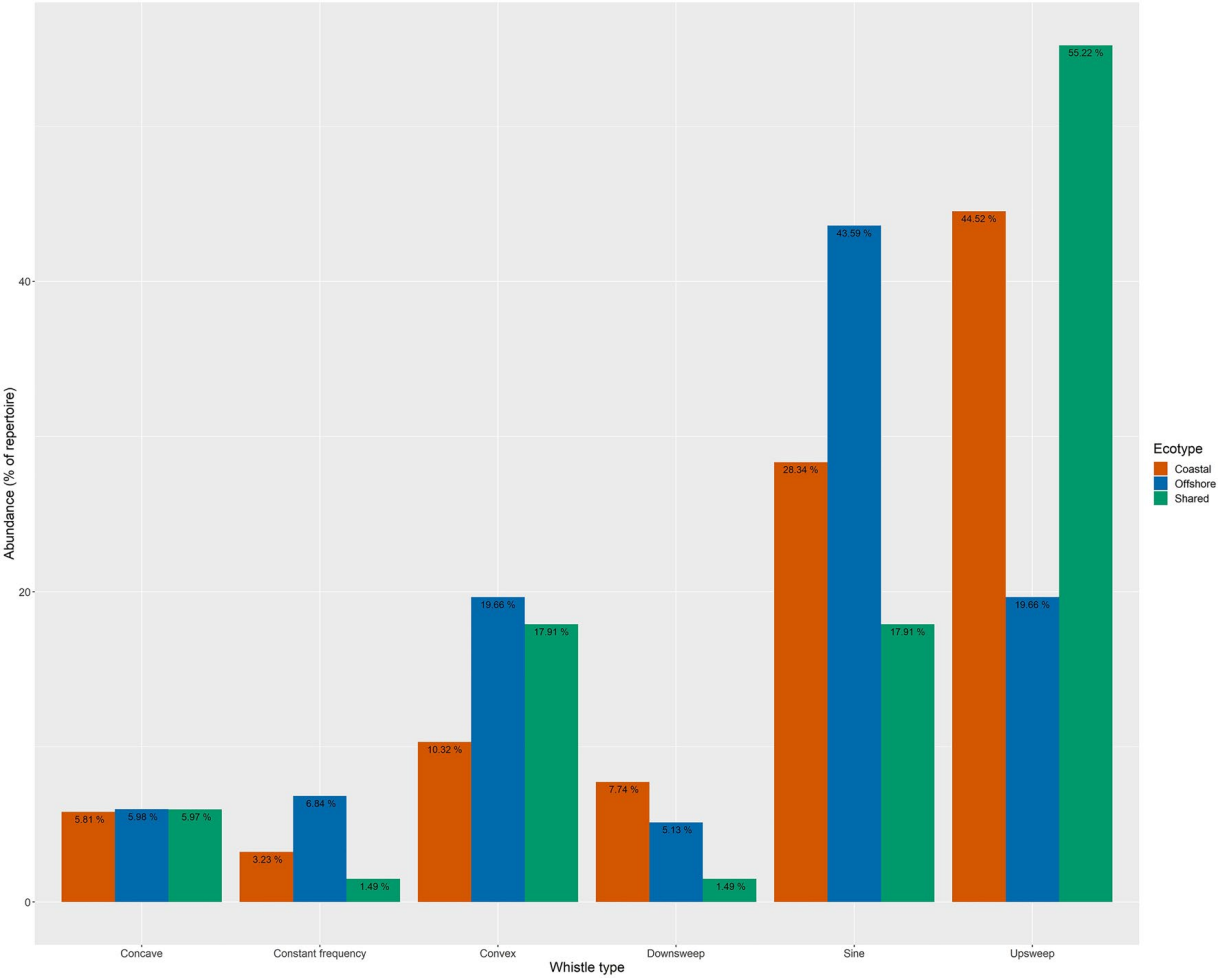
Overall abundance of pantropical spotted dolphin whistles per contour type within ecotype repertoires and the categories that were shared between ecotypes based on ARTwarp categorization of individual ecotype and combined datasets.

Whistles from both ecotypes were analyzed together through ARTwarp (n = 513 coastal whistles, n = 403 offshore whistles), resulting in 223 whistle categories. Within this species-wide repertoire, 86 categories were unique to the coastal ecotype, 70 to the offshore ecotype, and 67 were shared between ecotypes (Fig. 3). When ecotype datasets were categorized separately, ARTwarp categorized 681 coastal whistles (the full dataset) into 155 categories and 404 offshore whistles into 117 categories (Supp. Figs. S4, S5). These category counts are only used to compare general composition of contours and not used to compare diversity due to the large difference in sample size. Overall, both spotted dolphin ecotypes produced whistles with sine, upsweeps, downsweeps, constant frequency, convex, and concave contours (Fig. 4). However, in general upsweep (45%) and sine (28%) were most abundant in the coastal dolphin repertoire while sine (44%), upsweeps (20%), and convex (20%) contours were most common in the offshore dolphin repertoire (Fig. 4). Furthermore, most categories that were shared across ecotypes were upsweeps (55.22%) followed by sine and convex contours (17.91%).

**Figure 5:**
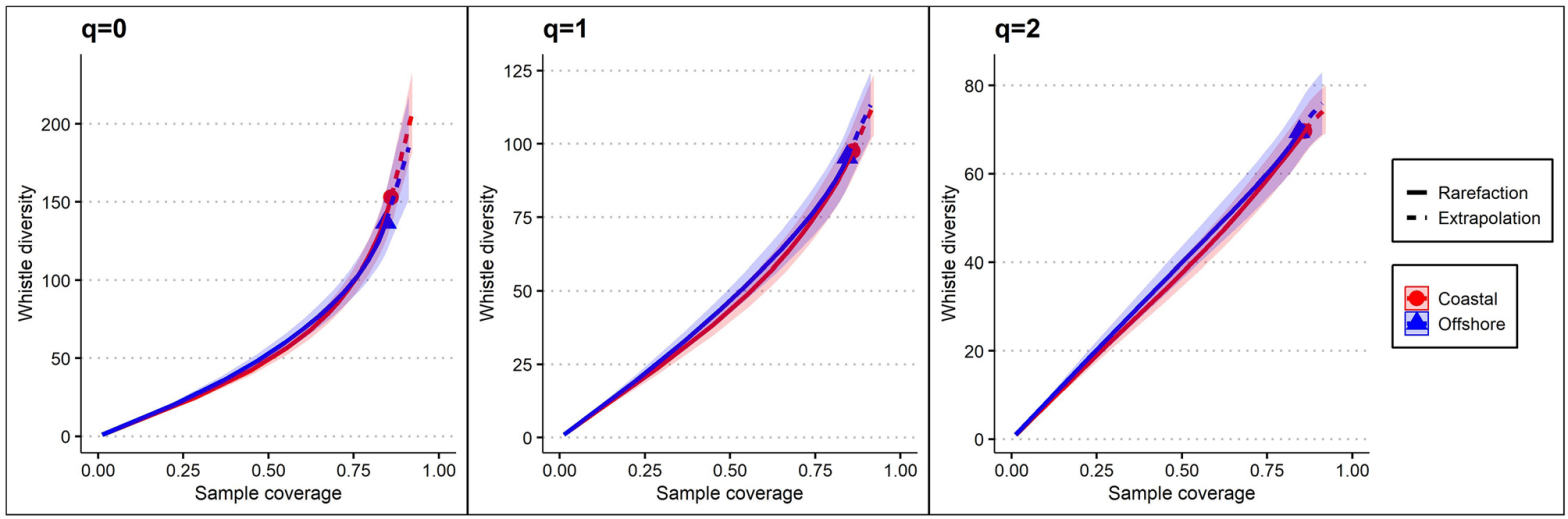
Rarefaction/extrapolation coverage-based curves for Hill numbers q = 0, 1, 2 of the offshore and coastal dolphin whistle repertoires in which data was extrapolated within an 84% CI. Due to overlap of the 84% CI’s, there is no biologically significant difference in repertoire diversity seen between ecotype repertoires.

Repertoire diversity and richness based on equal sample coverage of 90.25% were greater in the offshore ecotype. Whistle contour category richness estimates were 178.29 for the offshore ecotype and 190.51 for the coastal ecotype. The Shannon and Simpson diversity estimates for whistle contour categories in the offshore ecotype were 111.00 and 75.28 respectively, and 108.71 and 73.41 for the coastal ecotype (Table 3, Fig. 5). However, 84% confidence intervals of rarefaction and extrapolation curves overlap, indicating that this difference in effective number of whistle categories is biologically insignificant^41^.

**Table 3:** Asymptotic estimates of dolphin whistle category richness (q = 0), Shannon Diversity (q = 1) and Simpson Diversity (q = 2) of coastal and offshore dolphin whistle repertoires based on equal sample coverage of 90.25% and upper (UCL) and lower confidence limits (LCL).

Hill number	Ecotype	Estimate	LCL	UCL
q = 0	Offshore	178.29	142.02	214.56
	Coastal	190.51	151.17	229.86
q = 1	Offshore	111.00	94.11	127.90
	Coastal	108.71	95.44	121.97
q = 2	Offshore	75.28	64.09	86.48
	Coastal	73.41	65.77	81.06

The permutation test showed that the proportion of distinct ecotype whistle categories (69.95%) was significantly greater than expected based on random chance (mean_permuted_ = 50.09%; two-tailed test; α = 0.001, p = 0.0009). In measuring compositional similarity, the Horn Index, which is sensitive to dominant or common categories, in this case upsweep contours (Fig. 4), calculated a high compositional similarity of 64.73% (± 0.08, Fig. 4). In contrast, the Morisita-Horn Index that is more sensitive to rare categories estimated a low compositional similarity of 36.44% (± 0.04). The Morisita-Horn estimate of low compositional similarity refers to the difference in the distribution of whistle types within each repertoire as shown in Fig. 4. The differences between these indices illustrate the complexity of comparing dolphin whistle repertoires.

## Discussion

Our results show that coastal and offshore ecotypes of pantropical spotted dolphins in the Eastern Tropical Pacific have whistle repertoires that vary in acoustic structure and composition. This suggests that genetic distinctions^27,29,30^ and phenotypic specializations to their habitat^31^ could extend to their acoustic repertoire. Similar patterns have been described in coastal and offshore common bottlenose dolphins (*Tursiops truncatus*) in New Zealand and Mexico, with whistle repertoires of similar composition but varying acoustic structure^42,43^.

The observed variation in whistle frequency between pantropical spotted dolphin ecotypes could result from differences in the acoustic properties of their habitat. Coastal pantropical spotted dolphins live in acoustically dynamic environments due to high marine biodiversity, habitat complexity (i.e., coral reefs, mangroves, estuaries)^44–46^ and human presence (e.g., boat traffic)^33^. Coastal habitats are often shallow, hindering signal propagation due to transmission losses to the water surface and seafloor^33^. These properties of the coastal environment can negatively impact the communication range of coastal pantropical spotted dolphins, and result in selection for signals that propagate more successfully in noisier and more complex environments. For example, several studies have found that in shallow waters, coastal bottlenose dolphins from Florida^46^, Baja California, Mexico^43^, Bocas del Toro, Panama^47^, New Zealand^42^, and Southern Brazil^48^, and Indo-Pacific dolphins from Japan^34^ increased their communication range by producing low-frequency whistles. In contrast, in offshore habitats where the loss of acoustic energy is reduced, dolphins produced primarily higher frequency whistles. Similar patterns have been described for common bottlenose dolphins in New Zealand^42^, Croatia^35^, Baja California, Mexico^43^ and Brazil^48^. These results agree with our findings from the Random Forest analysis that find duration and frequency as the acoustic variables with the greatest impact on accurate classification, and therefore the variables that are most distinct between the ecotypes. Furthermore, the results of this study show within-ecotype variation in whistle fundamental frequency, some of which could be explained by the large geographical distance between the offshore groups. However, the MRPP and Multivariate Dispersion analysis show that there was significantly more within- and across-group variation in whistle fundamental frequency between ecotypes than between offshore groups and between coastal groups (Supp. Table S3). Overall, dolphins show a great degree of plasticity in their whistle frequency range, which allows them to quickly respond to changes in their acoustic environments^5,49,50^.

In addition to the acoustic properties of the habitat, differences in body size, group size, and behavioral activities could have contributed to the observed whistle variation between ecotypes. Coastal spotted dolphins are larger than offshore dolphins, which could explain some of the variation in whistle frequency^28^. However, in toothed whales, only 26% to 28% of the variation in whistle frequency is explained by body size, suggesting that other factors may be more important in explaining differences in signal frequency^51,52^. Overall, the evolutionary history of toothed whale whistle frequency is thought to be intertwined with social factors such as group size, as toothed whale species that live in large groups tend to communicate using higher frequency whistles than those living in smaller group sizes^52,53^. Because coastal pantropical spotted dolphins form smaller groups than their offshore counterparts^26^, some of the variation in whistle acoustic structure could be associated with group size, as has been reported in common dolphins^11^. However, establishing the contribution of group size to variation in whistle frequency is difficult, because group size is influenced by several factors including behavioral activities, food availability, social interactions, and predator presence^54^. These factors can also influence the type^55–57^ and acoustic structure of dolphin whistles^58,59^. The recordings used for this study did not have behavioral data associated with it, and therefore we could not measure its contribution. However, given that several studies have found that dolphin whistles can be highly context dependent^42,53,60^ future studies on whistle acoustic structure should take into account its contribution to whistle variation.

Regarding their whistle repertoire diversity, we find that pantropical spotted dolphin coastal and offshore ecotypes have similarly diverse whistle repertoires but vary in their whistle repertoire composition. We expected offshore dolphins to have a more diverse whistle repertoire than coastal dolphins because they live in larger groups. However, coastal pantropical spotted dolphins have very fluid and interchanging social group composition and are a highly abundant dolphin species in Central America^32^. As a result, it is possible that our recording effort included several distinct combinations of dolphin groups capturing high levels of whistle diversity. Another explanation may be related to their repertoire’s contour composition. Upsweeps contours were the most shared whistle type between the two ecotypes and the most used whistle type of the coastal ecotype. This result disagrees with previous contour analysis of pantropical spotted dolphins that found convex contours most common^25^ but follows the trend found in a variety of other delphinid species^61–63^. The offshore ecotype’s repertoire showed a higher presence of modulated whistle types such as sine, and to a lesser degree concave and convex (Fig. 4). Our study suggests that offshore dolphins have a greater use of frequency modulated contours than coastal dolphins, and we are interested in determining if other offshore pantropical spotted dolphin populations show similar levels of modulation. Frequency modulation patterns in dolphins can serve for species identification^16,64^, carry information about individual identity^65^, express emotional state during social interactions^5,66^, and vary with different acoustic environments^8,49^. Therefore, the observed differences in the proportion of modulated whistles in the repertoire of coastal and offshore dolphins could indicate population-specific differences in group size, social dynamics, and habitat specialization.

The offshore ecotype’s higher abundance of modulated whistles also correlates directly with the significant difference in whistle duration found between ecotypes. The data shows that the offshore ecotype produces significantly longer duration signals than the coastal ecotype. Dolphin whistle duration has been positively correlated with individual whistle contour complexity, likely a reflection of a more complex communication network in these larger social groups^5,15^. In spinner dolphins (*Stenella longirostris*), Guiana dolphins (*Sotalia guianensis*), and common bottlenose dolphins, whistle duration has been linked to surface behaviors such as anthropogenic interactions, foraging, socializing or resting^53,58,59,61^. Our data suggests that duration in addition to whistle modulation, may be linked to social organization and whistle frequency adaptations for better propagation and reception of signals.

In conclusion, this study finds evidence of ecotype acoustic distinction that may contribute to or reinforce the divergence of these dolphin lineages via habitat specialization and consequently, in the mediation and maintenance of group size and social dynamics. While repertoire diversity is conserved between ecotypes, repertoire composition, and frequency and duration characteristics of whistle contours are plastic and likely reflect local adaptations to a coastal and offshore lifestyle. Quantifying the vocal repertoire of pantropical spotted dolphins, is undoubtedly challenging, yet such characterization represents an important first step in understanding how these animals organize socially, and how they respond to changes in their environment. We have addressed this challenge by using methods that account for sampling size limitations, to provide the first characterization of this dolphin species’ whistle repertoire diversity. Future studies can expand upon this work by testing hypothesis on the role of group size and behavioral context on whistle repertoire size and composition. As anthropogenic climate change increases the speed of sound in the oceans^67^, this acoustic plasticity may prove to be crucial in adapting to increasingly noisier habitats. Finally, the combined analysis of whistle acoustic structure and repertoire features represents a novel approach for consideration in the development of dolphin species and population classifiers for passive acoustic monitoring programs.

## Methods

### Study area

This study took place in coastal and offshore waters of the ETP (Supp. Fig. S1). Recordings of the coastal ecotype were collected during boat-based research surveys focused on humpback whales in Padre Ramos, Northern Nicaragua and San Juan del Sur, Southern Nicaragua. In El Salvador, acoustic recordings were made as part of Energía del Pacífico, Ltda. de C.V. (“Energía del Pacífico”) Marine Biodiversity Monitoring and Evaluation Program. (MBMEP). Recordings were obtained from Metalío to the northern part of Punta Remedios within the Los Cóbanos Natural Protected Area. Here, boat surveys were completed across a total of 10 perpendicular transects. In both countries boat surveys were conducted using a small boat 7–10 m in length and a 60 HP engine. Boat surveys were from 7 a.m. to 4 p.m. and when dolphins were detected, information about group size and behavior, and acoustic data were collected. In both sites recordings were made using a Zoom Recorder with a sampling rate of 44.1 kHz using an Aquarian Scientific hydrophone model AS-1 (linear range: 1 Hz—100 Hz ± 2 dB; sensitivity − 208 dBV re ± 2 dB).

Recordings of the offshore ecotype were collected as part of United States National Oceanic and Atmospheric Association (NOAA) cetacean abundance research cruises. The *Stenella* Abundance Research (STAR) surveys of 2000 and 2006 covered the area from the United States-Mexico border, south to the territorial waters of Peru and west to Hawaii^68,69^. The Hawaiian Island and Ecosystem Assessment Survey (HICEAS) of 2002 took place in the United States Exclusive Economic (EEZ) of Hawaii from the island of Hawaii to the Kure Atoll in the northwest^70^. The 2005 Pacific Islands Cetacean Ecosystem Assessment Survey (PICEAS) recorded in the United States EEZ of the Palmyra Atoll, Kingman Reef, and Johnson Atoll, in addition to the waters between these EEZ’s and the Hawaiian Islands^71^. Surveys were completed during daylight hours on predetermined line-transects. Researchers estimated school size and identified species using 25 × 150 high power binoculars from the ship’s flying bridges^68^. Cetacean vocalizations were detected with hydrophone arrays that were towed at a depth of 6–11 m and between 200 and 300 m behind the research vessel. The STAR2000 cruise used an array of 5 hydrophones with a frequency response of 15 Hz − 40 kHz ± 4 dB at − 132 dB re 1 V/mPa^65^. The HICEAS2002 cruise used an array of 3 hydrophones with a frequency response of 500 Hz  − 25 kHz ± 10 dB at − 155 dB re 1 V/mPa^70^. The PICEAS2005 cruise used an array of 3 hydrophones with a frequency response of 1 kHz − 40 kHz ± 5 dB at − 150 dB re 1 V/mPa^71^. The STAR2006 cruise used an array of 2 hydrophones with a frequency response of 1 kHz − 40 kHz ± 5 dB at − 150 dB re 1 V/mPa^69^. Vocalizations were recorded on Tascam DA-38 (STAR2000, HICEAS 2002, STAR2006) and Tascam DA-78 (PICEAS2005) multi-channel recorders with a sampling rate of at least 96 kHz (Supp. Table S1).

### Whistle data collection

We used RAVEN PRO 1.5 build 37^72^ to create spectrograms of each recording with a fast Fourier transform (FFT) size of 1,024 points, an overlap of 50%, and using a 512-sample Hann window. Selections were made with a border of 0.5 s to ensure that no contour was cut off during extraction. Whistle detection was performed manually, and selection was based on the following rules: whistles had a clear and dark contour from start to end (Signal-to-Noise Ratio above 4 dB); whistles were considered individual if separated by at least 200 ms from other whistles in the recording; overlapping whistles were selected only if clearly distinguishable from one another; and whistles in a recording were selected to maximize representation of different types of contours^61^. Given that acoustic recordings were made at different sampling rates, to standardized whistle selection we only used whistles with fundamental frequency contours below 22.05 kHz.

Whistle selections were uploaded into Luscinia (https://github.com/rflachlan/Luscinia) for manual contour tracing and Beluga (https://synergy.standrews.ac.uk/soundanalysis/) for automated contour extraction. Luscinia is a semi-automatic contour analysis software in which spectrograms are uploaded and contours can be manually traced to extract acoustic parameters for analysis^73^. The following standard settings were used during contour tracing: frame length (ms) = 5, tie step (ms) = 1, spectrographic points = 221, spectrographic overlap% = 80, dynamic range (dB) = 82, dereverb range (ms) = 50, windowing function = Gaussian, frequency zoom = 100, NR range1 (ms) = 50, NR range2 (ms) = 50. Dynamic range (dB) was used as a second assessment that all selections had adequate signal to noise ratio. Dynamic range adjusts the gray scale within the spectrogram and specifies the threshold after which point pixels are rendered as white and unable to be traced in Luscinia. In Luscinia, the following standard acoustic variables (e.g., Refs.^2,8,74^) were extracted from each whistle: minimum frequency (MF) (measures the frequency in the lowest point in the contour), maximum frequency (MXF) (measures the frequency at the highest point in the contour), start frequency (SF), end frequency (EF), duration (D), delta frequency (DF) (this is the difference between MF and MXF) and peak frequency (PF) (frequency where the maximum amplitude occurred) (Supp. Fig. S2). Beluga was used to extract contours for use in ARTwarp^75^ (https://synergy.st-andrews.ac.uk/soundanalysis/). The following standard settings were used during contour extraction in Beluga: Extraction method = peak frequency; FFT length = 2048; frame length = 512; frame overlap = 87%; harmonics not included.

### Statistical analysis

Acoustic parameter measurements from Luscinia were exported into R^76^ to compile descriptive statistics. A Mann–Whitney U test (α = 0.05) with Bonferroni correction in R was employed to determine whether there was a significant difference in the acoustic parameter measurements between ecotypes^76^. Only offshore ecotype whistles with a maximum frequency of less than or equal to 22.05 kHz (466/653 whistles) were used for analysis to be consistent with the lower sampling rate used in coastal surveys.

#### Comparison of fundamental whistle contours

To have comparable sample sizes between ecotypes, a random subsample of the coastal dataset was created by omitting every 4th whistle within the dataset. Luscinia’s built-in dynamic time-warping (DTW) function was used to analyze the distribution of fundamental frequency contours based on measurements of time, fundamental frequency, fundamental frequency change and vibrato amplitude (the amplitude a signal’s rapid modulation, or “buzz”). These features were established as most important for the analysis of contour similarity within a dataset first in birds, then in dolphins^42,75,77^. Fundamental frequency and fundamental frequency change (the slope of the spectrograph at a given point, either increasing, decreasing, or remaining constant) have been deemed crucial to include because delphinids are known to perceive both relative and absolute frequency changes^78^. Vibrato amplitude was included as a measure of periodic oscillations within a contour. These contour features were normalized relative to each other by calculating the standard deviations of each parameter. Weightings used in the DTW were: Time = 10.0 ms; Fundamental frequency = 3.513; Fundamental Frequency Change = 2.413; Vibrato Amplitude = 1.973. DTW compresses or expands the time domain of spectrograms to maximize the frequency overlap of whistles being compared and calculates a dissimilarity score based on the contour features that were weighted for each whistle contour (compiled in a dissimilarity matrix). Based on what is known about signature whistle use and recognition, dolphins are known to be relatively insensitive to variation in signal duration and more sensitive to changes in frequency and therefore DTW allows for the comparison of contours that have similar modulation patterns with variation in duration^79^.

After DTW, we performed a two-dimensional NMMDS analysis and a k-medoids clustering analysis in Luscinia based on the dissimilarity matrix that was produced during DTW. NMMDS presents a scatter plot of the relative distance (based on (dis)similarity) between the sample whistles as calculated for the distance matrix. NMMDS visualizes the distribution of each dataset, and how their distributions cluster relative to each other with labelled data. Luscinia’s k-medoids cluster analysis assessed natural clustering of the species-wide dataset based on fundamental frequency contour using unlabeled data.

To ensure that an ecotype comparison was appropriate, we used Multivariate Response Permutation Procedure (MRPP) tests and multivariate dispersion tests in Luscinia to further explore the variation within and between offshore and coastal groups. Because of insufficient sample size, the offshore group from NOAA Cruise STAR2006 and the coastal group from Padre Ramos, Nicaragua were not included in these analyses (Supp. Table S1). The MRPP shows how closely each offshore, coastal, or ecotype group’s dataset is clustered. First, the mean distance between data points (whistle contours) is calculated from the dissimilarity matrix and then the empirical delta test statistic is calculated as the mean of these distances. Next, the data is permuted 10,000 times to produce the predicted delta test statistic. The MRPP provided the empirical and predicted delta test statistics, a p-value (the proportion of permutations that resulted in predicted delta values equal or smaller than the empirical delta value), and an effect size a-value. Effect size ranges from 0 to 1 where an a-value of 0 means that the groups exhibit the level of variability expected by chance, and an a-value close to 1 means that there is no variation within the group. A multivariate dispersion analysis used the dissimilarity scores from the dissimilarity matrix to compare the variability of whistle contours measured within each group and compares this group variability across groups using the Levene’s F-statistic. Dissimilarity scores were permuted 10,000 times and the significance of the differences between groups is assessed by the F-statistic and p-value. In this manner, multivariate dispersion analysis provides an independent comparison measure of whistle contour composition between groups.

#### Classification of whistles into ecotypes

Random forest (RF) classification determined the viability of, and variable importance for, distinguishing between coastal and offshore ecotypes based on whistle parameter measurements. This analysis was performed using the *randomForest* package in R^76,80^. RF classification is a non-parametric analysis that uses an ensemble of decision trees to categorize data based on predictor variables^81^. Each decision tree takes a bootstrapped sample of the dataset, uses 2/3 to grow the tree, and saves 1/3 as an out-of-bag (OOB) sample to assess the classification accuracy of that tree. A random selection of predictor variables is considered at each node within the tree to partition the data in a way that maximizes the homogeneity of the following nodes. The final classification of each whistle is based on the majority vote of all trees in the model. Acoustic datasets of the acoustic variables used in the RF analysis were exported from Luscinia. A random subsample of whistles was taken from the coastal ecotype dataset to match the 466 whistle offshore ecotype sample size to ensure that the classification was not skewed^80^. The ecotype datasets from Luscinia were combined into a species-wide dataset and split into training (67%) and testing (33%) with an equal distribution of each ecotype in each dataset. Pearson’s correlation coefficients were calculated for each variable to ensure that overfitting did not occur due to correlation between variables^82^. The acoustic variables measured in Luscinia were used as the random forest’s predictor variables. All Pearson correlation coefficients fell between ± 0.8 indicating that they were uncorrelated and therefore independent enough to be included in the model^83^. The optimal number of random predictor variables (mtry) considered at each node was tuned using repeated k-fold cross-validation and the optimal mtry was determined by the model with the largest area under the receiver operating characteristic (ROC) curve. We used Mean Decrease in Accuracy (MDA) and Mean Decrease in Gini (MDG) to assess variable importance. MDA gives the mean normalized measure of the loss in prediction performance if a variable is permuted^81^. The MDG gives a measure of how much each variable plays a role in the homogeneity of the nodes. For both measures, a higher value indicates higher importance.

A confusion matrix of the RF classification results, and Cohen’s Kappa statistic were used to evaluate the model’s performance. The confusion matrix displayed the number of correctly and incorrectly classified whistles for each ecotype. Cohen’s Kappa statistic is a measure of the observed accuracy (the RF classification results) compared to the expected accuracy (random chance) and is an accepted method evaluating machine learning classifiers^81^.

#### Whistle repertoire complexity and diversity

We assessed contour repertoire diversity using ARTwarp to categorize contours extracted using Beluga. ARTwarp is a MATLAB program that uses DTW to compare whistle contours and automatically categorizes contours based on contour similarity using an unsupervised adaptive resonance theory neural network, the ART2 neural network^79^. The ART2 algorithm compares input whistle contours to a set of reference whistle contours using DTW, and either determines the inputs to be similar enough to a reference whistle contour to be grouped with it, or dissimilar enough to warrant a new reference category. This decision point is based on a user-determined vigilance parameter that was set to 96%. A 96% vigilance was used based on previous literature which showed that this vigilance level successfully categorized bottlenose dolphin signature whistles^79^. Though it is unknown whether pantropical spotted dolphins have signature whistles, 96% vigilance was used to not discount their possible existence. Each reference contour is an amalgamation of all the whistle contours in that category. Reference contours are updated each time a new whistle is added to a category. In this way, the reference categories continuously update based on the dataset. ARTwarp was used to categorize whistles from each ecotype dataset individually. The ecotype datasets were then combined to compare ecotype repertoire diversity.

Ecotype repertoires and the whistle contour categories that the ecotypes shared were categorized further into general contour categories following Bazua and Au^61^ contour classification through visual inspection. Whistle reference categories were classified as ascending if increasing in frequency without inflection points, descending if decreasing in frequency without inflection points, convex if increasing in frequency and then decreasing in frequency with an inflection point, concave if decreasing in frequency then increasing in frequency with an inflection point, sine if multiple inflection points, and constant if there was a change in frequency less than or equal to 1 kHz (Supp. Fig S6).

To analyze the diversity of repertoires as categorized by ARTwarp, an asymptotic estimate of species richness (in this case, whistle richness), Shannon diversity and Simpson diversity with Hill numbers was used^84^. Hill numbers provided the effective number of whistle categories based on varying sensitivity to rare categories (q = 0, 1, 2). Effective number of categories refers to the number of categories with equal abundance needed to get the same diversity measure^84^. R/E curves were plotted based on sample completeness (Supp. Fig. S7). Sample completeness is measured by sample coverage which is the proportion of individuals (whistles) in the assemblage that belong to a category represented by the dataset. In other words, it is the proportion of whistles that belongs to a category represented by the dataset as opposed to a category that the dataset did not account for Ref.^85^. This method accounts for the inevitable failed detection of all categories that exist. In comparing the repertoire diversity of coastal versus offshore ecotypes, the coverage-based R/E sampling curves were analyzed at up to double the sample size of the smaller dataset (the offshore dataset) (as per Ref.^84^). Estimates of species richness, Shannon Diversity and Simpson Diversity were compared at a sample coverage of 90.25%.

To compare the composition of repertoires between ecotypes, a permutation test was performed based on the ARTwarp output of the combined ecotype dataset. This test was used to determine whether the proportion of ecotype specific categories (ARTwarp categories that contained whistles produced by only one ecotype) was significantly greater than the expected proportion given a repertoire with no ecotype distinction. A total of 1000 permutations were performed by randomly resampling categories and determining the proportion of categories within samples that were ecotype specific. A two-tailed test was used to calculate whether there was a significantly (< 0.001) greater proportion of ecotype specific categories in the dataset than expected. Repertoire (dis)similarity was further explored using the SpadeR package in Rstudio (v0.1.1; Ref.^86^) to calculate pairwise similarity statistics. Abundance-based Horn and Morisita-Horn index measures were calculated to account for the relative abundance of whistles in each category and difference in sample size^87^.

### Supplementary Information

Supplementary Information.

## Data Availability

Data and scripts will be made available upon request to Laura May-Collado at lmaycoll@uvm.edu.
