# Supplementary material for: Whistle repertoire and structure reflect ecotype distinction of pantropical spotted dolphins in the Eastern Tropical Pacific
Source: Sci Rep. 2023 Aug 18;13:13449. doi: 10.1038/s41598-023-40691-8 (PMC10439233; doi:10.1038/s41598-023-40691-8)
Supplement: Supplementary file 1 — Supplementary Information. [file 41598_2023_40691_MOESM1_ESM.pdf]

Whistle repertoire and structure reflect ecotype distinction of pantropical spotted dolphins in the Eastern Tropical Pacific

Manali Rege-Colt[1], Julie N. Oswald[2], Joelle De Weerdt[3,4], Jose David Palacios-Alfaro[5], Maia Austin[1], Emma Gagne[1], Jacqueline Maythé Morán Villatoro[6], Catherine Teresa Sahley[7], Gilma Alvarado-Guerra[8], and Laura J. May-Collado[1,9 *]

[1]University of Vermont, Biology Department, Burlington, VT, United States
[2]Scottish Oceans Institute, Sea Mammal Research Unit, University of St. Andrews, St. Andrews, KY168LB, UK
[3]Association ELI-S, Education, Liberté, Indépendance - Scientifique, Allée de Verdalle 39, 33470 Gujan-Mestras, France
[4]Vrije Universiteit Brussel (VUB), Pleinlaan 2, 1050 Brussels, Belgium
[5]Panacetacea.Org, Saint Paul, MN, United States
[6]Energía del Pacífico, Ltda. de C.V. /Invenergy LLC; Municipality of Acajutla, Sonsonate, El Salvador
[7]Environmental Resources Management, Inc., Breckville, Ohio, United States
[8]Instituto para el Crecimiento Sostenible de la Empresa (ICSEM), C/Hogar Padre Vito Guarato, B1, EL Salvador
[9]Smithsonian Tropical Research Institute, Panama, Panama
Corresponding author: [*]lmaycoll@uvm.edu

Supplemental Material

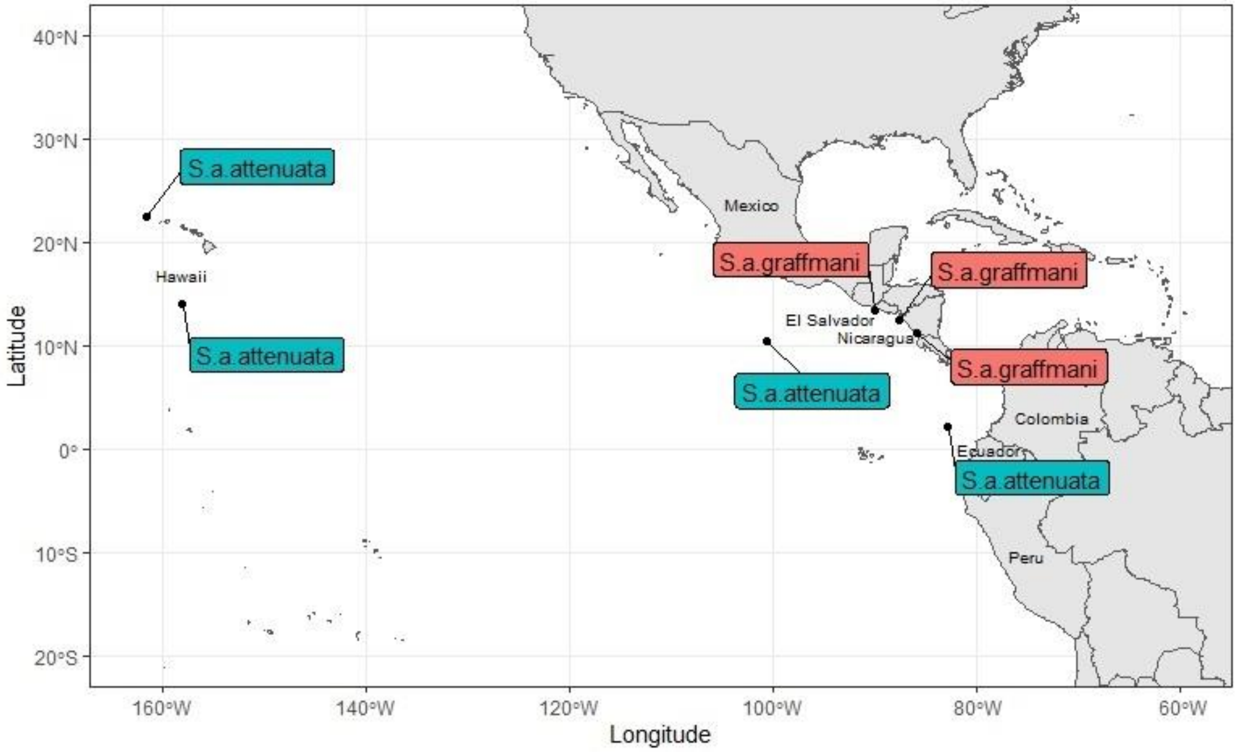

Supplementary Figure S1. Map of locations where recordings were taken. Recordings of the

coastal ecotype (*S.a. graffmani*) were collected from El Salvador and two locations in Nicaragua (Padre Ramos and San Juan del Sur) from over-the-boat hydrophones. Offshore ecotype (*S.a. attenuata*) recordings were collected from four areas in the Eastern Tropical Pacific using towed hydrophone arrays.

Supplementary Table S1. Sampling effort for recordings analyzed in this study. The table shows information on the location and dates of field work as well as the recording sampling rate, total number of whistles used, number of groups of dolphins recorded, and the number of individuals within those groups.

Dataset	Location/cruise	Field period	Sampling rate (kHz)	Sampling effort (hours)	Whistle contour sample size	# Groups	Min-max group size
**Coastal ecotype**	Los Cóbanos Ntl. Park, El Salvador	12/11/20-03/18/2021	44.1	1:58:07	322	20	1-20
	Padre Ramos, Nicaragua	04/07/17-04/15/2018	44.1	0:27:51	68	9	10-40
	San Juan del Sur, Nicaragua	09/12/2017-2/25/2020	44.1	1:58:15	267	17	4-150
**Offshore ecotype**	STAR2000	07/28/2000-12/09/2000	150	0:39:06	148	5	37-360
	HICEAS2002	07/27/2002-12/09/2002	150	0:37:54	206	5	19-80
	PICEAS2005	07/28/2005-11/29/2005	96	3:33:30	298	6	92-209
	STAR2006	07/28/2006-12/07/2006	192	0:10:00	3	1	148

Supplementary Table S2. Descriptive statistics of pantropical spotted dolphin's contour frequency and temporal variables by ecotype. A Mann-Whitney U test (α=0.05) was performed to compare whistle variables between ecotypes (using data with sampling rates less than or equal to 44.1 kHz). Statistically significant differences between ecotypes are represented by "*".

**Ecotype (Sampling rate)**	**Measure**	**Max freq. (kHz)***	**Min freq. (kHz)***	**Start freq. (kHz)***	**End freq. (kHz)***	**Delta freq. (kHz)**	**Peak freq. (kHz)***	**Duration (s)***
**Coastal ecotype (44.1 kHz) (n = 492)**	Mean (±sd)	16.00 (±3.44)	7.16 (±2.42)	8.49 (±3.54)	14.85 (±4.22)	8.83 (±4.20)	9.51 (±2.70)	0.61 (±0.36)
	CoV	21.49%	33.83%	41.69%	28.39%	47.57%	28.36%	58.72%
	Max	21.96	18.69	21.56	21.96	19.26	19.29	2.70
	Min	6.53	1.65	1.66	3.87	0.09	1.97	0.02
**Offshore ecotype (44.1 kHz) (n = 466)**	Mean (±sd)	17.62 (±2.98)	8.85 (±2.26)	10.75 (±3.80)	13.80 (±4.60)	8.77 (±3.50)	11.53 (±2.37)	0.91 (±0.41)
	CoV	16.90%	25.52%	35.34%	33.29%	39.93%	20.57%	44.70%
	Max	21.96	14.50	21.96	21.96	17.67	20.32	4.19
	Min	5.68	3.12	3.12	3.28	0.31	5.27	0.06

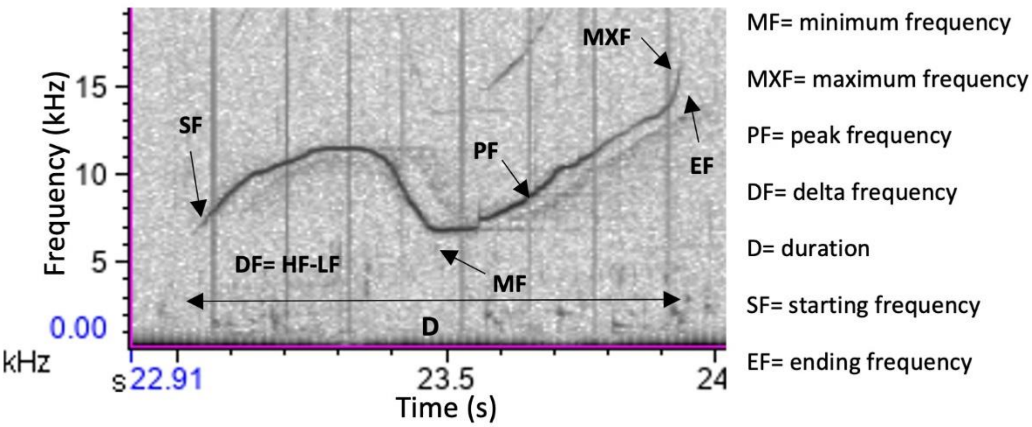

Supplementary Figure S2. Example of a spectrogram showing a whistle's frequency and relative amplitude over time. Standard acoustic parameter measurements are highlighted and were extracted from each whistle in Luscinia.

Supplementary Table S3. Results of multivariate response permutation procedure (MRPP) and multivariate dispersion analysis analyses carried out with offshore, coastal, and ecotype datasets. MRPP and multivariate dispersion analyses investigate within and between group whistle contour variation based on dissimilarity matrices of whistles that have undergone Dynamic Time Warping.

Groups	Analysis	Test Statistic
**Offshore**	MRPP	p = 0.0001
		a = 0.0383
		empirical δ = 0.235
	Multivariate Dispersion	p = 0.836
		F = 0.176
**Coastal**	MRPP	p = 0.0001
		a = 0.00901
		empirical δ = 0.245
	Multivariate Dispersion	p =0.464
		F = 0.530
**Ecotype**	MRPP	p = 0.0001
		a = 0.0493
		empirical δ = 0.244
	Multivariate Dispersion	p = 0.0046
		F = 8.364

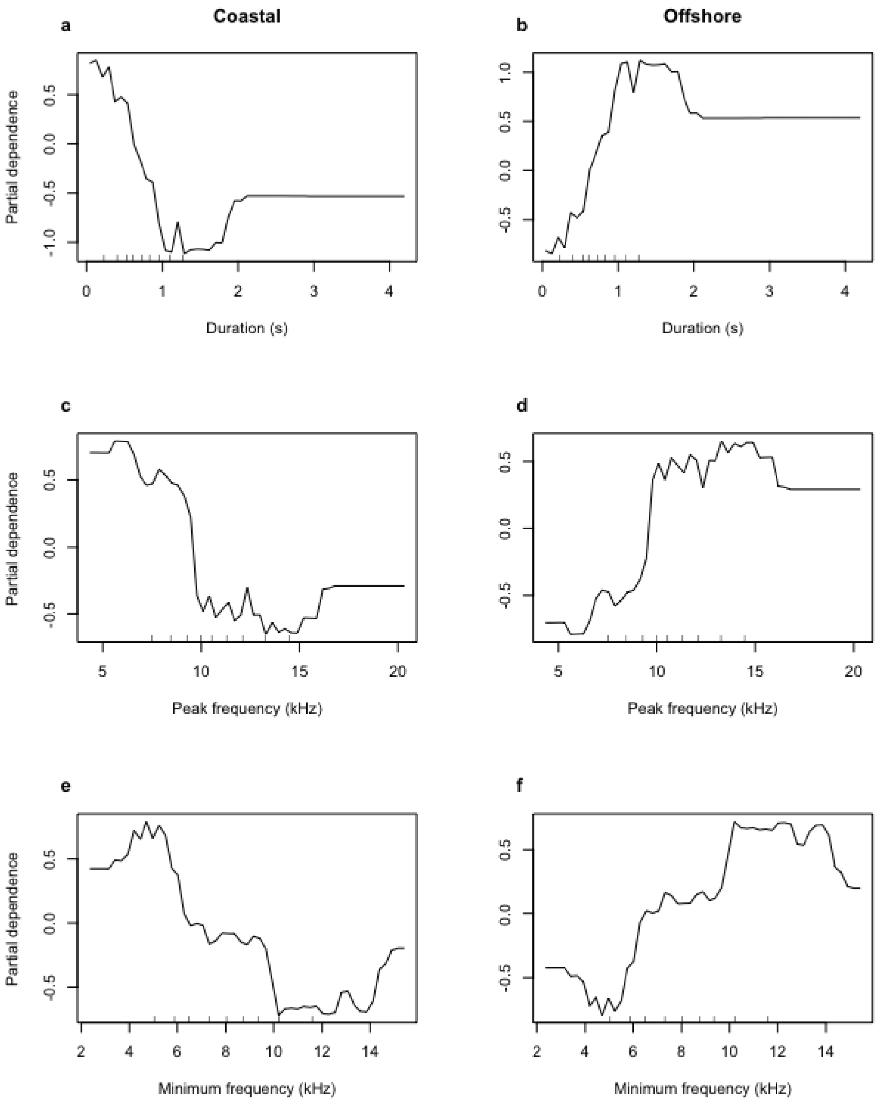

Supplementary Figure S3. Partial dependence plots of the acoustic parameters that best predicted ecotype in the random forest analysis based on MDA variable importance. Classification by duration (a, b), peak frequency (c, d) minimum frequency (e, f) is shown. Partial dependence, or the impact of the variable of interest on classification accuracy is found on the y-axis with partial dependences above 0 having impact.

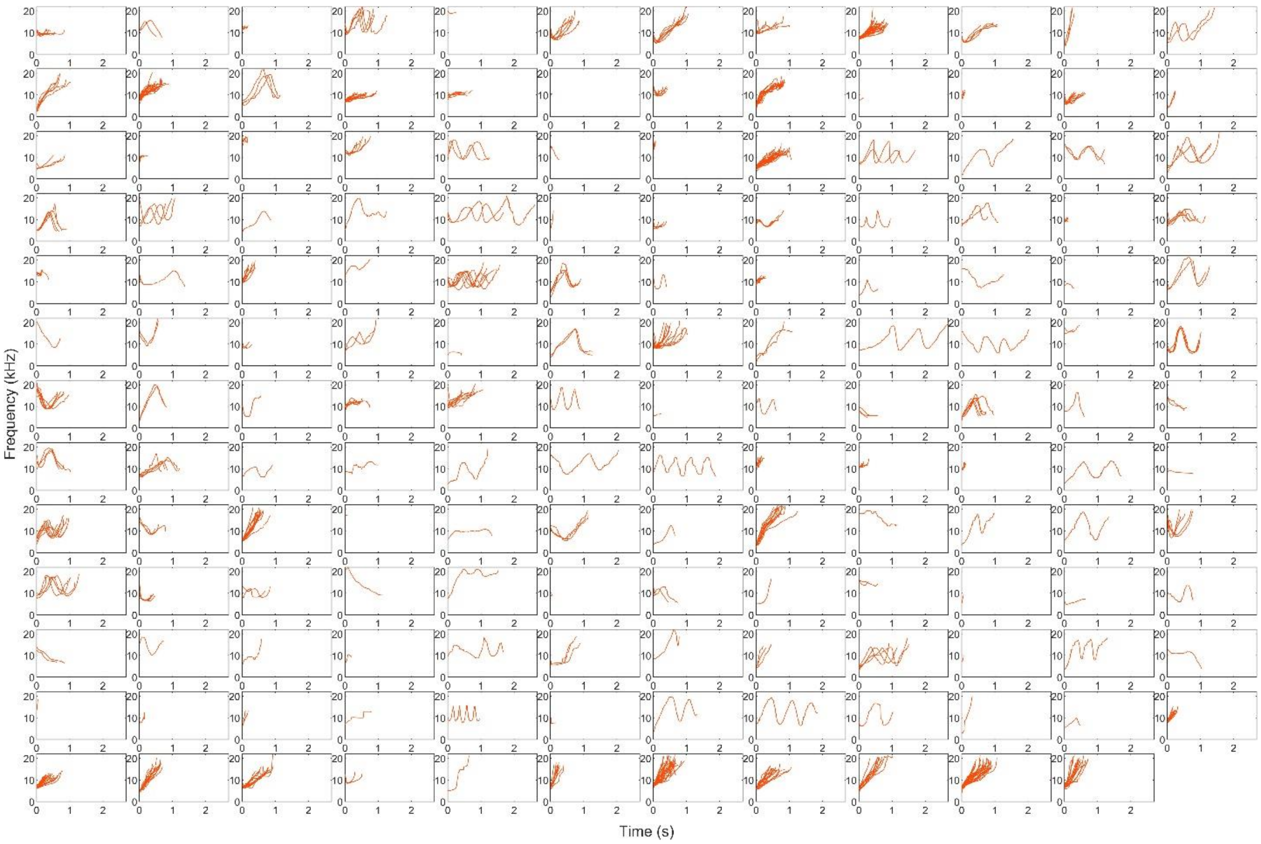

Supplementary Figure S4. The ARTwarp categorization of the coastal dataset (n=681) using a vigilance of 96%. The categorization resulted in 155 categories. Each window is a neuron that represents the spectrogram of a category that the neural network has created with time on the x-axis (0-5 seconds) and frequency on the y-axis (0-20 kHz). Within each neuron are the contours that were categorized as belonging to that neuron.

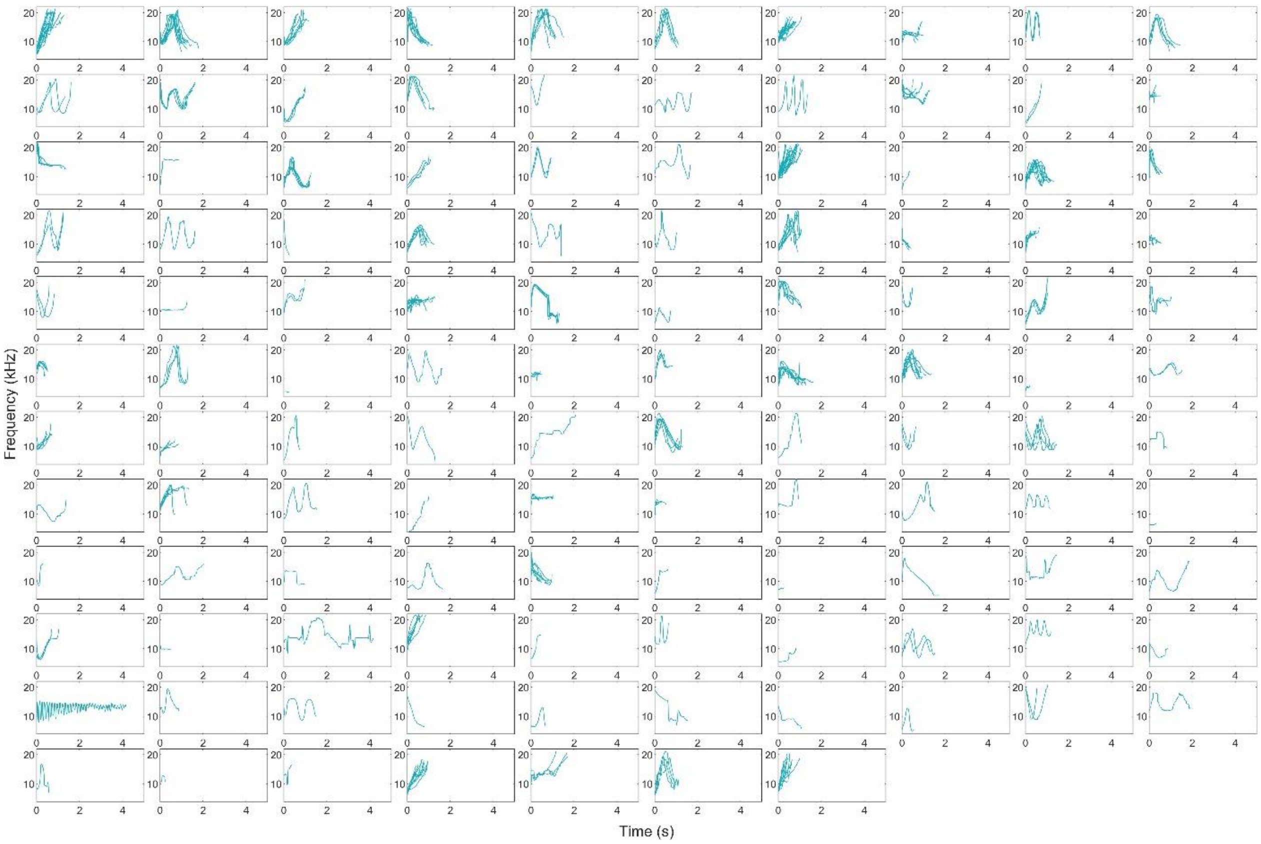

Supplementary Figure S5. The ARTwarp categorization of the offshore ecotype whistles (n=403) using 96% vigilance. The categorization resulted in 117 whistle categories. Each window is a neuron that represents the spectrogram of a category that the neural network has created with time on the x-axis (0-5 seconds) and frequency on the y-axis (0-20 kHz). Within each neuron are the contours that were categorized as belonging to that neuron.

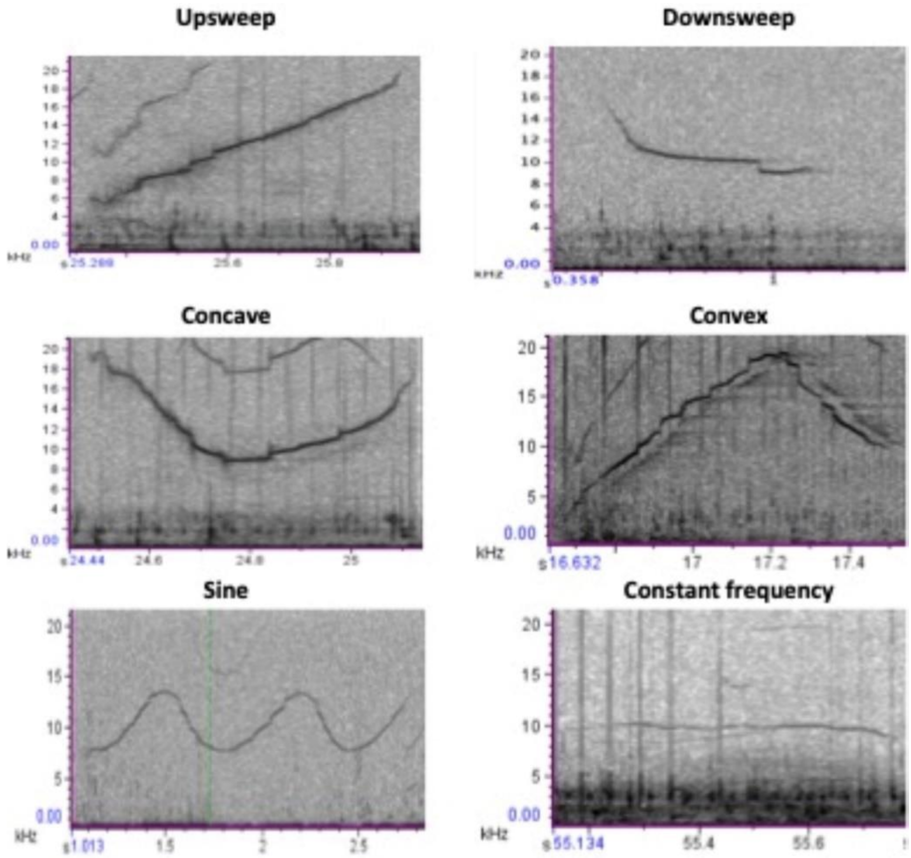

Supplementary Figure S6. Whistle contours based on Barzúa-Durán and Au (2002), used in this study to classify the whistle contour category outputs from ARTwarp for each ecotype's repertoire and the combined categorization.

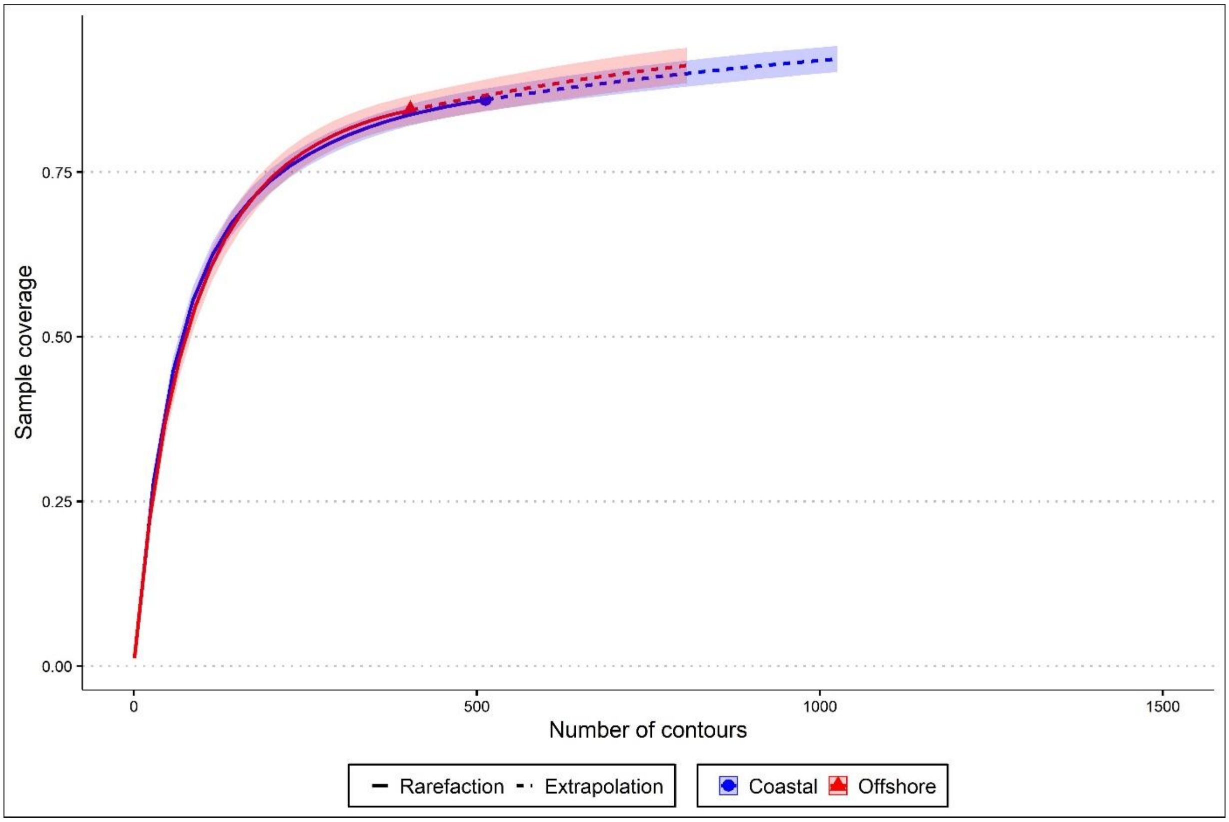

Supplementary Figure S7. Rarefaction/Extrapolation curve showing sample completeness of the coastal and offshore ecotype datasets based on their respective sample sizes of 526 and 444 whistles with 84% confidence intervals.
